# Gross motor function, functional level, physical performance, and muscle strength in preschool children newly diagnosed with cancer: Data from the RePlay trial

**DOI:** 10.1007/s00431-026-07333-3

**Published:** 2026-08-18

**Authors:** Anna Pouplier, Jan Christensen, Peter Schmidt-Andersen, Helle Winther, Hanne Bækgaard Larsen, Martin Kaj Fridh

**Affiliations:** 1 Department of Pediatric and Adolescent Medicine, Juliane Marie Centre, Copenhagen University Hospital - Rigshospitalet, Blegdamsvej 9, DK-2100 Copenhagen, Denmark; 2 Department of Occupational Therapy and Physiotherapy, Copenhagen University Hospital - Rigshospitalet, Copenhagen, Denmark; 3 Department of Clinical Medicine, Faculty of Health and Medical Sciences, University of Copenhagen, Copenhagen, Denmark; 4 Department of Nutrition, Exercise and Sports, Faculty of Science, University of Copenhagen, Copenhagen, Denmark

**Keywords:** Preschoolers, Cancer, Gross motor function, Functional level, Physical performance, Handgrip strength, Newly diagnosed

## Abstract

Children with cancer experience treatment-related physical disruptions; however, these disruptions for preschool children newly diagnosed with cancer are poorly characterized. This study aimed to quantify gross motor function, functional level, physical performance, and muscle strength in preschool children aged 1–5 years newly diagnosed with cancer, and to compare these with age-expected normative values. This cross-sectional study includes data from the baseline assessment of 83 children (49% girls, 39 ± 16 months old) enrolled in the Rehabilitation including structured active Play for preschoolers with cancer (RePlay) trial. Gross motor function was evaluated using the Peabody Developmental Motor Scales–Second Edition (PDMS-2); functional level was assessed using the Pediatric Evaluation of Disability Inventory (PEDI); physical performance was assessed using the 6-min walk test; and muscle strength was assessed using the KLS Martin Vigorimeter for handgrip strength. Mean gross motor quotient was 78.9 [95% CI: 73.6–84.2], indicating poor overall performance; 76% of the children performed below average or worse. Most children had difficulties with locomotion, with a mean standard score of 5.7 [95% CI: 4.8–6.6], indicating poor performance; 81% of the children performed below average or worse. PEDI mobility data were available for 83 children, with a mean normative standard score of 23.2 [95% CI: 20.0–26.3], indicating decreased functional level; 69% of the children scored below average. Mean handgrip strength was below average compared to healthy children; 0.17 [95% CI: 0.12–0.22].

*Conclusion*: These results show that preschoolers newly diagnosed with cancer have gross motor function, functional level, and muscle strength impairments, underlining the importance of targeted physical rehabilitation. *ClinicalTrials.gov*: NCT04672681.

**Table:** 

**What is Known:** *• Research has shown that children newly diagnosed with cancer have markedly impaired muscle strength, cardiorespiratory fitness, physical performance, and gross motor functions*. *• Gross motor function development is fundamentally important for preschoolers. However, research on gross motor function in children with cancer is limited, and existing studies often include broad age ranges (1–18 years), hindering clear evidence specific to preschoolers aged 1–5 years*.
**What is New:** *• Preschool children newly diagnosed with cancer have clinically significant impairments of gross motor function and functional level*. *• Data on physical performance and muscle strength are limited, indicating poor feasibility for preschoolers with cancer*.

**Supplementary Information:**

The online version contains supplementary material available at https://doi.org/10.1007/s00431-026-07333-3

## Introduction

Children diagnosed with cancer are at risk of developing multiple treatment-related physical side effects [1, 2]. The combined impact of chemotherapy, radiotherapy, glucocorticoid therapy, and prolonged sedentary behavior during hospitalization, including bed rest and reduced participation in leisure activities, has been shown to affect the central and peripheral nervous systems as well as muscle morphology, thereby limiting physical activity levels [2, 3]. Among the most studied treatment-related complications is chemotherapy-induced peripheral neuropathy, which is characterized by sensory impairments such as numbness, paresthesia, and burning sensations [2, 4]. Studies involving children with cancer aged 1–17 years have demonstrated reduced and delayed muscle action potentials associated with neuropathy, resulting in impaired muscle function [5, 6]. In addition to neuromuscular impairments, children with cancer aged 4–18 years exhibit significant reductions in upper- and lower-extremity muscle strength compared with healthy age- and sex-matched peers, with deficits evident even during the early stages of treatment [7–10]. Furthermore, cardiorespiratory fitness has been reported to be significantly compromised in children aged 6–18 years at diagnosis, throughout treatment, and up to one year following treatment completion when compared with healthy controls [11–13]. Collectively, impairments in muscle function, muscle strength, and cardiorespiratory fitness contribute to reduced motor performance, including deficits in coordination, balance, and movement speed [14–16]. For preschoolers, impairments in gross motor function are of particular interest, as appropriate gross motor development is essential for overall development in early childhood [17–19] and for future physical activity [17, 18]. The evidence on early gross motor function impairments is limited to a few studies that include only children with acute lymphoblastic leukemia across a broad age range of 1–18 years [1, 19, 20], hindering clear evidence on preschoolers specifically. Determining the prevalence and magnitude of impairments in gross motor functions and functional level in preschool children newly diagnosed with cancer is fundamental for informing early identification and developing supportive rehabilitation strategies. Therefore, this study aimed to quantify gross motor function, functional level, physical performance, and muscle strength in preschool children aged 1–5 years newly diagnosed with cancer and to compare these with age-expected normative values.

## Method

This study is reported in accordance with the STROBE (STrengthening the Reporting of Observational Studies in Epidemiology) statement for cross-sectional studies [21].

### Design and setting

This is a cross-sectional study based on baseline assessment data collected between 2021 and 2024 from the Rehabilitation including structured active Play for preschoolers with cancer (RePlay) trial (NCT04672681) at the Department of Pediatric and Adolescent Medicine at Copenhagen University Hospital — Rigshospitalet in Denmark. The trial investigated the effectiveness of a 6-month structured active play intervention on gross motor function development. A detailed description of the trial is published in the study protocol [22].

### Study population

Children newly diagnosed with cancer (i.e., all cancer diagnoses), between 1 and 5 years of age, treated with intravenous chemotherapy and/or radiotherapy, with parents who could communicate in Danish, were eligible for the study. Children diagnosed with mental disorders that prevent them from following instructions in relation to the intervention and testing were excluded.

### Study procedure

Children were enrolled in the RePlay trial within 14 days of treatment initiation. Upon enrolment and prior to randomization, a physical assessment was scheduled to assess 1) gross motor function, 2) muscle strength, and 3) physical performance. Assessment of gross motor function was prioritized. The aim was to schedule and complete the physical assessment within 14 days of treatment initiation. However, assessments conducted up to 4 weeks after treatment initiation were accepted. Although the assessment could be divided across multiple sessions, if necessary, this was never required in practice. Scheduling a single assessment session within the first month was often challenging, and additional sessions were generally considered unlikely to improve assessment completion rates. All assessments were conducted in the hospital's pediatric physiotherapy clinic by the primary investigator (AP), in collaboration with an experienced pediatric physiotherapist. Parents were present during the assessment and were encouraged to support and motivate their child during testing procedures. In addition, parents completed a proxy-reported outcome measure to evaluate their child’s functional level.

### Measures

#### Gross motor functions

To assess gross motor functions, we used the gross motor subset of the Peabody Developmental Motor Scales—Second Edition (PDMS-2) for children aged 0–6 years [23]. The subtest includes three domains: stationary (e.g., getting up from the floor, balancing on knees, balancing on one leg, standing on tiptoes) (30 items), locomotion (e.g., crawling, walking, running, jumping, walking up and down stairs, walking on a line, jumping on one foot) (89 items), and object manipulation (e.g., throwing, catching, and kicking different sized balls) (24 items). Each item is given a score of 0, 1, or 2.

The test is administered by starting with a given item in the domain, depending on the child’s age in months. The items are treated singularly until the child scores 2 on three consecutive items, which determines the lower level of performance. It may be necessary to revert to earlier items to achieve three consecutive scores of 2. Earlier items, prior to the lower level, are all given a score of 2. The test then proceeds to further items until the child scores 0 on three consecutive items, thereby determining the ceiling level. The remaining items above the ceiling level are all given the score of 0. The item scores are summed to obtain a raw score for each domain, which is then converted to an age-adjusted standard score and percentile rank for each domain. The standard score is summed to create a gross motor quotient and percentile ranks. The test has shown acceptable psychometric properties, including Cronbach α = 0.89–0.97, test–retest *r* = 0.82–0.93, and interscorer *r* = 0.96–0.99 [23].

Normative data are available for children aged 0–6 years [23]. For the individual domains, standard scores are between 1–20. Standard scores are interpreted relative to a normative mean of 10 and a standard deviation of 3, where 6–7 (9th-16th percentile) indicate “below average” performance, scores 4–5 (2nd-5th percentile) indicate “poor” performance, and scores 1–3 (below 2nd percentile) indicate “very poor” performance. Gross motor quotient scores are between 38–165. Gross motor quotients are interpreted relative to a normative mean of 100 and a standard deviation of 15, where 80–89 (9th-23rd percentile) indicates “below average” performance, 70–79 (2nd-8th percentile) indicates “poor” performance, and 35–69 (below 2nd percentile) indicates “very poor” performance [23].

#### Functional level

Functional level was assessed using the Pediatric Evaluation of Disability Inventory (PEDI) for children aged 6 months to 7.5 years old [24]. The assessment instrument is a proxy measure of the capability and performance of selected functional activities and was administered as a structured interview with parents. The PEDI includes three scales: functional skills, caregiver assistance, and modifications. The functional skills scale is divided into three domains: self-care (e.g., eating, tooth brushing, showering, going to the toilet, blowing your nose, putting on clothed) (73 items), mobility (e.g., getting on and off a chair/toilet, getting in and out of a bed and bath, moving around indoors and outdoors, getting up and down stairs) (59 items), and social function (e.g., the ability to understand and communicate, playing with peers and adults, problem solving, own safety, orientation in time and daily activities) (65 items), with each item scored with a 0 or 1. The caregiver assistance scale is divided into three domains: self-care (8 items), mobility (7 items), and social function (5 items), with each item scored 0 to 5. For this study, we only analyze the data from the mobility domain of the functional skills scale and the caregivers’ assistance scale.

The items in each scale are summed to a raw score for each domain, which is then converted into an age-adjusted normative standard score. The Danish version of the PEDI has been tested, with item difficulty found to be comparable (*r* = 0.82–0.97) on all six subscales and intra-respondent reliability found to be good (intraclass correlation coefficient = 0.62–0.97) [25].

Normative data are available for children aged 6 months to 7.5 years [24]. Normative standard scores provide a measure of the child’s functional performance and caregivers’ assistance needs within a domain relative to his/her peers. For the individual domains, normative standard scores are interpreted relative to a mean of 50 and a standard deviation of 10. A normative standard score of 30 is two standard deviations below the normative mean, and 95% of children would typically perform at a higher level. For interpretive purposes, we indicate a normative standard score of ≤ 30 as “below average,” as scores below 30 indicate functional delays, since children without disabilities generally have scores between 30 and 70 (50 ± 2 SD) [25]. A normative standard score of 10 is four standard deviations below the normative mean, and 99% of children would typically perform at a higher level. Scores outside the 10–90 range are given a normative standard score of “below 10” or “above 90”.

#### Physical performance

The 6-min walk test (6MWT) is a general physical performance indicator [26]. The test was conducted in a hallway with a 15-m track clearly marked at each end. The child was asked to walk from one mark to the other as many times as possible within six minutes. The assessor verbally encouraged the child to keep walking. Parents could walk beside their child along the track, but not holding hands, and the child decided the pace. Additionally, the distance walked after the initial two minutes was recorded in case the child did not complete all six minutes. The reference distance in meters (mean ± 1 SD) for healthy children aged 3–5 years from Geiger et al. (2006) was used for comparison: males, 536.5 ± 95.6; females, 501.9 ± 90.2 [27]. For interpretative purposes, we indicate a distance ≤ −1 SDS as “below average”.

#### Muscle strength

Muscle strength was measured using the KLS Martin Vigorimeter for handgrip strength [28]. The instrument is a pneumatic dynamometer with three sizes of rubber bulbs. The smallest bulb was used for all participants in this study. The bulb was placed with the air tube extending outward between the thumb and index finger, and the fingers wrapped around it. The test was conducted twice per hand, with left and right hands alternating, and a 30-s break between trials. The children were verbally encouraged to squeeze the bulb as hard as they could. The highest score for each hand was used for the analysis. Standard values for healthy children are set for the KLS Martin Vigorimeter between 0.1–0.4 bar [28]. The reference grip strength in bars (mean ± 1 SD) for healthy children aged 3–5 years from Robertson & Deitz (1987) was used for comparison. The reference grip strength was provided for age ranges of 6 months and for both right and left hands, with values of 0.23 ± 0.6 to 0.42 ± 0.9 for right-hand grip strength and 0.23 ± 0.5 to 0.40 ± 0.10 for left-hand grip strength [29]. For interpretative purposes, we indicate a value ≤ −1 SDS for the comparative age-group as “below average”.

#### Statistical analysis

Prior to statistical analysis, data quality control was performed using double data entry. Two researchers independently scored all PDMS-2 and PEDI records, and any discrepancies were resolved through consensus. Descriptive analyses were used to summarize and to compare gross motor function, functional level, physical performance, and muscle strength with age-expected normative values [23, 24, 27, 28]. For PEDI scores reported as “below 10”, a value of 10 was assigned. A sensitivity analysis was conducted using an alternative imputed value of 1 to assess the robustness. Between-group differences in gross motor function and functional level were analyzed using analysis of covariance (ANCOVA) models, allowing residual variance to differ by diagnostic group (hematological cancers, extracranial solid tumors, and central nervous system tumors). All models were adjusted for time since treatment initiation (days).

Associations between time since treatment initiation and outcomes were examined exploratively using linear regression. All analyses were performed in R (version 3.6.0) and RStudio. A two-sided p-value < 0.05 was considered statistically significant.

#### Ethics

Informed consent was obtained from parents prior to inclusion in the RePlay trial. The Replay trial is approved by the Danish Data Protection Agency (jr. Nr.: P-2020–290), and the Regional Research Ethics Committee in the Capital Region of Denmark (jr.nr.: H-20023949).

## Results

**Fig. 1:**
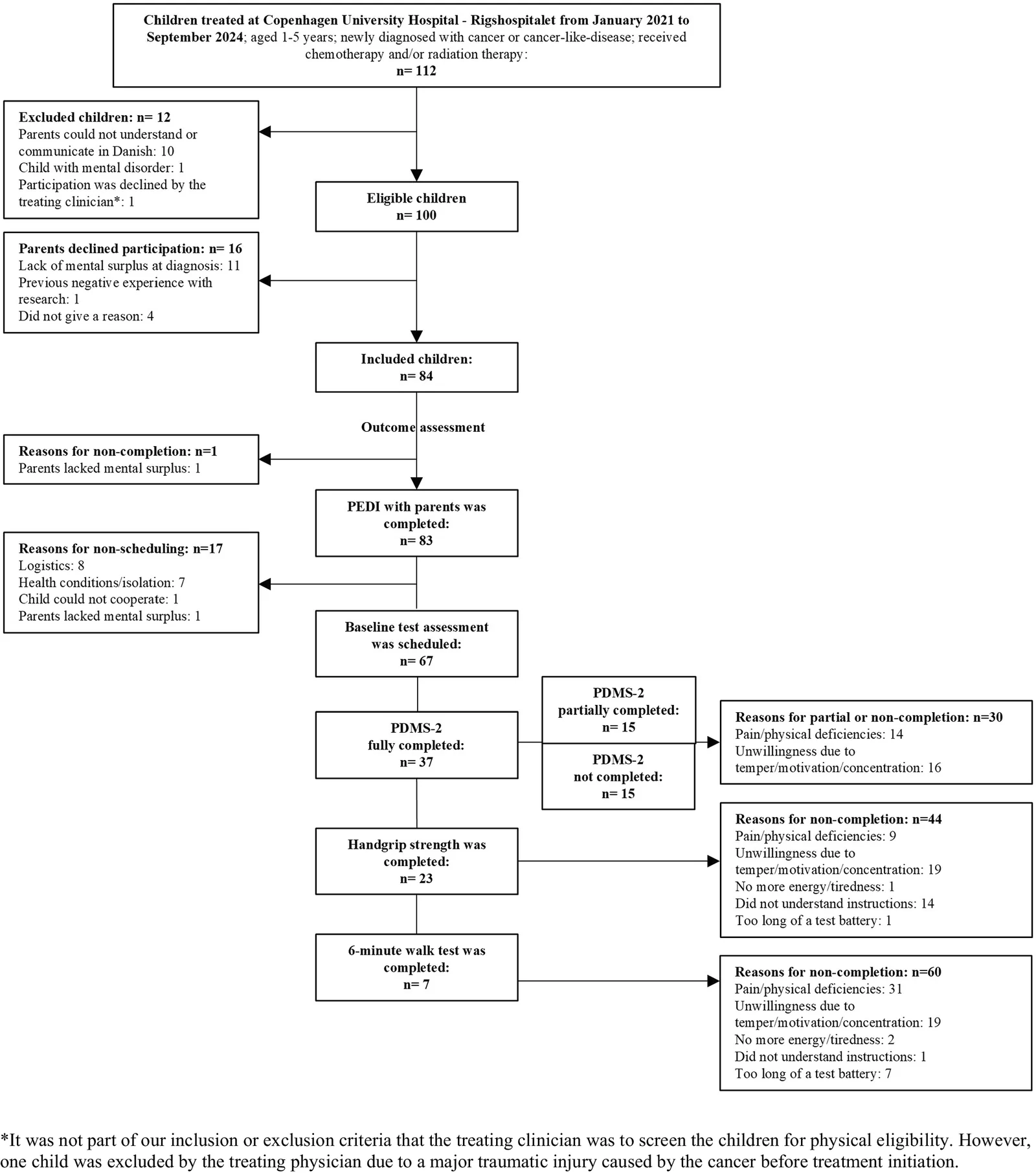
Flowchart of inclusion, scheduling of baseline test-assessment, baseline test-completion, and PEDI completion

Between 2021 and 2024, 84 of 100 eligible children (84%) with newly diagnosed cancer were enrolled, and 83 completed at least one of the outcomes at baseline. Participants' characteristics are presented in Table 1. Of the 83 children, we were able to schedule a baseline test-assessment with 67; of these, 37 fully completed the PDMS-2, 15 completed at least one domain, and 15 did not complete any domain. Additionally, 23 children (all of them > 3 years old) completed the handgrip strength test, and 7 children (6 of them > 3 years old, 1 of them < 3 years old) completed the 6-min walk test. A total of 83 parents completed the PEDI. Reasons for non-scheduling and non-completion are shown in Fig. 1. A thorough description of the feasibility of the PDMS-2, PEDI, handgrip strength, and 6-min walk test is published elsewhere [30]. On average, the physical assessments that were scheduled were conducted 13 days (0–27) after treatment initiation. At the time of the physical assessment, 99% had started Chemotherapy, and 11% had undergone surgery in the central nervous system. Additionally, 92% of the children had initiated treatment with Vincristine, and 75% with Dexamethasone (Table 1).

**Table 1:** Participant characteristics

Anthropometric characteristics	All children (*n* = 83)	Children who fully/partially completed the PDMS-2 (*n* = 52)	Children who did not schedule or complete the PDMS-2 (*n* = 31)	Children who completed the handgrip strength test (*n* = 23)	Children who did not complete the handgrip strength test (*n* = 60)
Sex (males/females)	41/42	23/29	18/13	10/13	31/29
Age in months, (SD)	39 ± 16	39 ± 16	39 ± 16	52 ± 11	34 ± 15
Height in cm, (SD)	99 ± 12	96 ± 12	105 ± 10	108 ± 7	96 ± 12
Weight in kg, (SD)	16 ± 4	15 ± 4	18 ± 3	19 ± 2	15 ± 4
Diagnostic group					
Hematological cancers	55 (66%)	32 (62%)	23 (74%)	15 (65%)	40 (67%)
Central nervous system tumors	13 (16%)	10 (19%)	3 (10%)	6 (26%)	7 (12%)
Extracranial solid tumors	15 (18%)	10 (19%)	5 (16%)	2 (9%)	13 (22%)
Treatment modalities*					
Chemotherapy (total planned/initiated)	82 (99%)/82 (99%)	51 (98%)/51 (98%)	31 (100%)/31 (100%)	23 (100%)/23 (100%)	59 (98%)/59 (98%)
Vincristine (total planned/initiated)	81 (98%)/76 (92%)	38 (73%)/35 (67%)	29 (94%)/27 (87%)	16 (70%)/15 (65%)	51 (85%)/47 (78%)
Dexamethasone (total planned/initiated)	64 (77%)/62 (75%)	48 (92%)/47 (90%)	30 (97%)/29 (94%)	23 (100%)/23 (100%)	55 (92%)/53 (88%)
Radiation therapy (total planned/initiated)	9 (11%)/0 (0%)	6 (12%)/0 (0%)	3 (10%)/0 (0%)	4 (17%)/0 (0%)	5 (8%)/0 (0%)
Surgery (total planned/initiated)	23 (28%)/9 (11%)**	16 (31%)/6 (12%)**	7 (23%)/3 (7%)**	8 (35%)/4 (17%)**	15 (25%)/5 (8%)**

*A child may have more than one treatment modality

**Only children with central nervous system tumors had undergone surgery prior to the baseline assessment

**Fig. 2:**
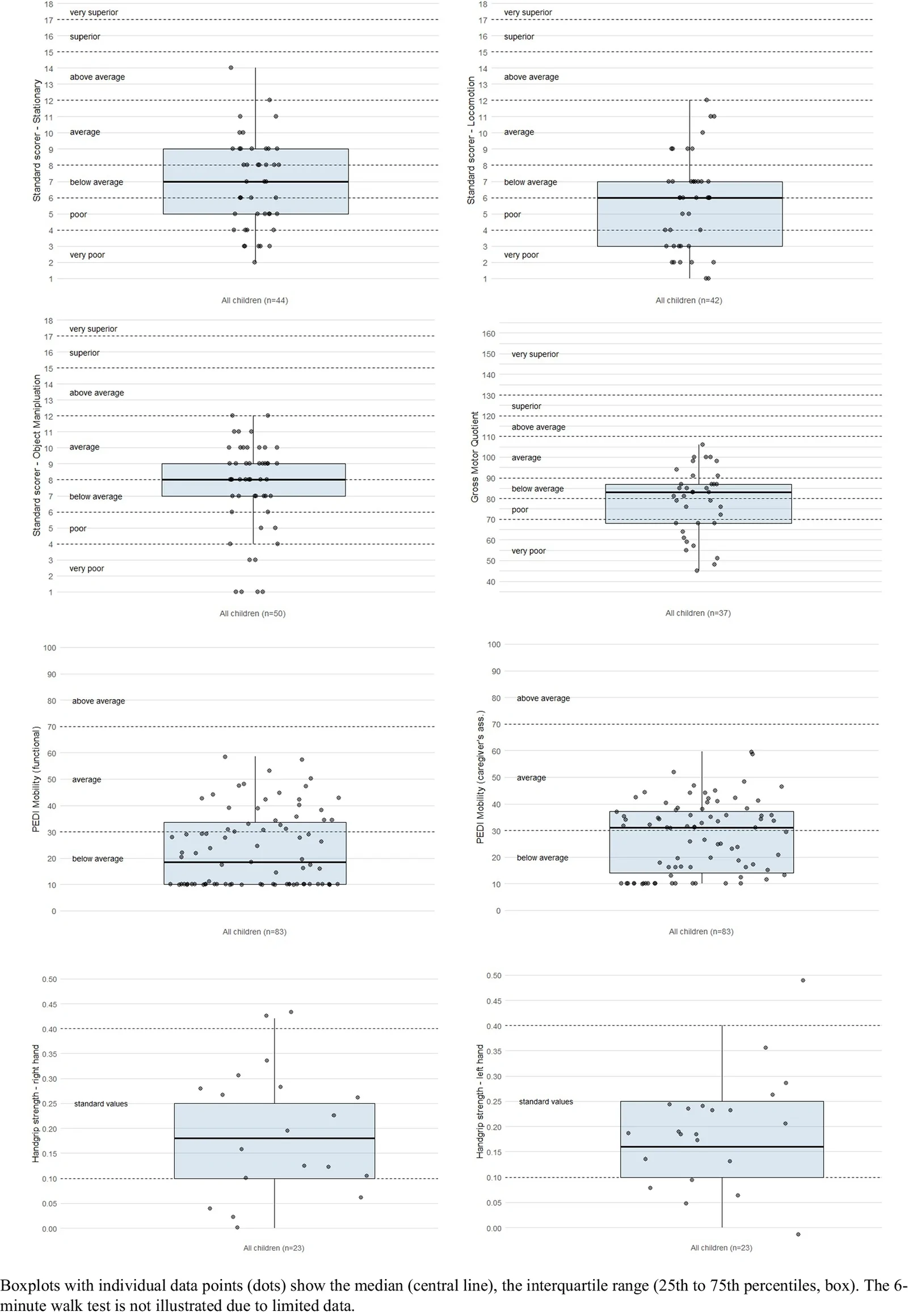
Distribution of standard scores and the gross motor quotient of the PDMS-2, the PEDI, and the handgrip strength

On the PDMS-2, children with cancer showed “below average” mean standard scores in stationary and object manipulation, and “poor” mean standard scores in locomotion and the gross motor quotient. The lowest mean standard score was in locomotion, where 81% of the children had scores below the 16th percentile, corresponding to a −1 standard deviation score (SDS) or worse from the normative mean of 10 ± 3 SD. In the functional mobility domain of the PEDI, 69% of children had a normative standard score below 30, corresponding to a −2 SDS or worse relative to the normative mean of 50 ± 10 SD. For handgrip strength, 79% of the children performed −1 SDS or worse below the reference mean for healthy children for right-hand grip strength and 83% for left-hand grip strength. Mean standard scores, gross motor quotient, bar, and meters, 95% CI, range, and percentage of children scoring below average or worse of the PDMS-2, the PEDI, handgrip strength, and 6-min walk test are shown in Table 2, and the distribution is illustrated in Fig. 2.

**Table 2:** Mean standard score and gross motor quotient, 95% CI, range, and percentage of children scoring below average of the PDMS-2 and the PEDI; mean bar, 95% CI, range, and percentage of children with handgrip strength below the standard values; and mean meters, 95% CI, range, and percentage of children with 6-min walking distance below the reference mean

	Mean	95% CI	Range	Children scoring below average or worse (%)^#^
PDMS-2—Stationary (*n* = 44)	6.8	6.0–7.7	2–14	24/44 (55%)
PDMS-2—Locomotion (*n* = 42)	5.7	4.8–6.6	1–12	34/42 (81%)
PDMS-2—Object Manipulation (*n* = 50)	7.6	6.7–8.3	1–12	19/50 (38%)
PDMS-2—Gross Motor Quotient (*n* = 37)	78.9	73.6–84.2	45–106	28/37 (76%)
PEDI Mobility (functional) (*n* = 83) “below 10” = 10	23.2	20.0–26.3	10–58.5	57^▪^/83 (69%)
PEDI Mobility (functional) (*n* = 83) “below 10” = 1	19.4	15.4–23.3		
PEDI Mobility (caregivers’ ass.) (*n* = 83) “below 10” = 10	27.4	24.5–30.4	10–59.6	40^▪▪^/83 (48%)
PEDI Mobility (caregivers’ ass.) (*n* = 83) “below 10” = 1	25.6	22.1–29.1		
Handgrip strength – right hand (*n* = 23)	0.17	0.12–0.22	0.00–0.42	18/23 (78%)
Handgrip strength – left hand (*n* = 23)	0.17	0.12–0.22	0.00–0.40	19/23 (83%)
6-min walk test (*n* = 6)*	347	265.5–428.5	255–480	5/6 (83%)

^#^ For the PDMS−2, this equals a score ≤ the 16th percentile/−1 SDS; for the PEDI, this equals a score ≤−2 SDS; for handgrip strength, this equals a value ≤−1 SDS; for the 6−min walk test, this equals a distance ≤−1 SDS

*Should be interpreted with caution due to very limited data. One child was removed from analysis as the child was 16 months old, and no reference data exists for children <3 years

^▪^35 of the 57 children had a normative standard score “below 10”; ^▪▪^ 17 of the 40 children had a normative standard score “below 10”

**Fig. 3:**
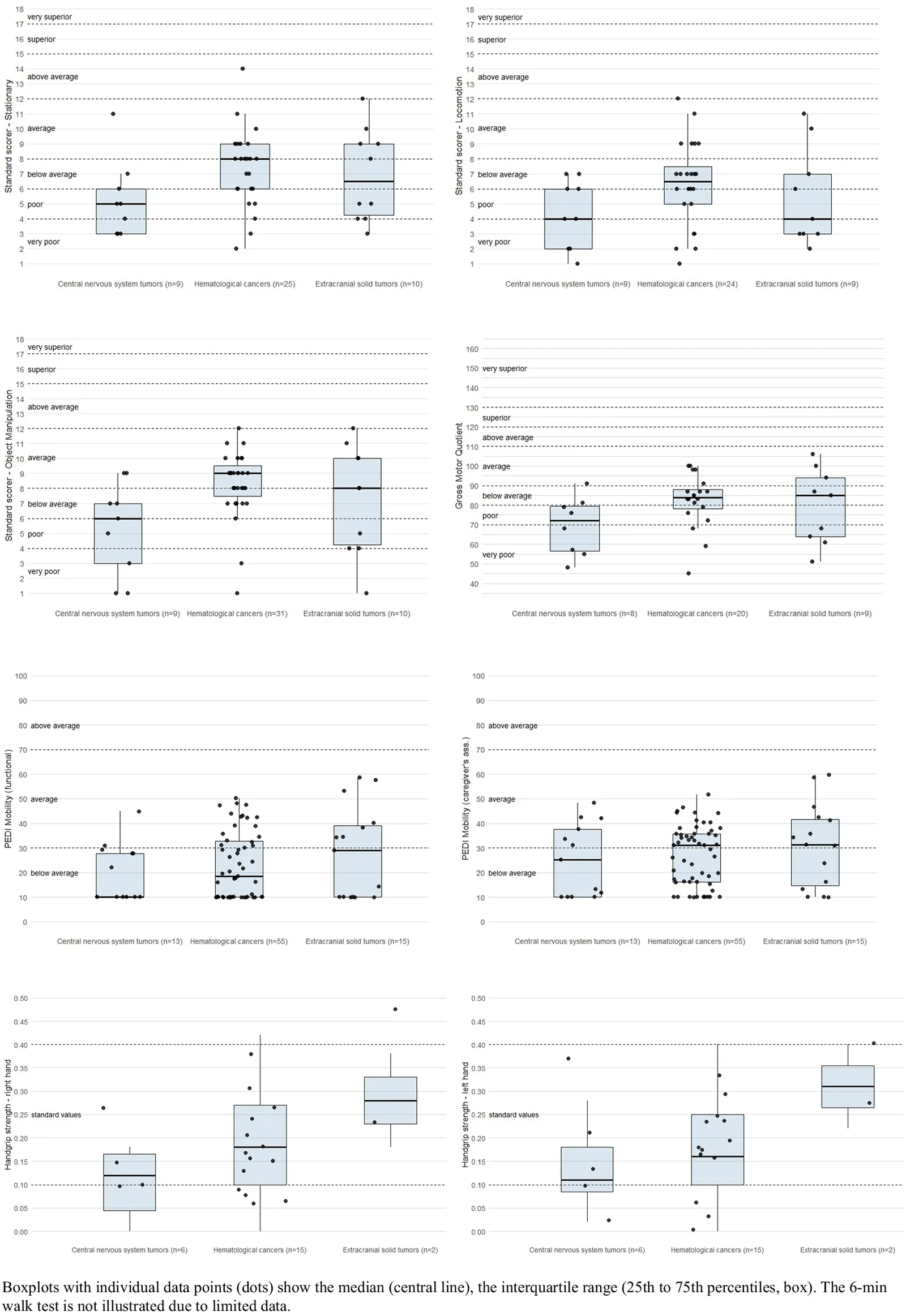
Distribution of standard scores and the gross motor quotient of the PDMS-2, the PEDI, and the handgrip strength

A significant difference in mean standard scores (−2.56 [95% CI: 0.51 to 4.61], p = 0.02) was found in the object manipulation domain of the PDMS-2 between children with hematological cancers and those with central nervous system tumors. No significant difference was found between the diagnostic groups in the stationary domain, locomotion domain, or gross motor quotient of the PDMS-2, or the mobility functional scale and caregivers’ scale of the PEDI (Supplemental Information (SI−1)). Between-group differences in handgrip strength were not analyzed due to small subgroups. No association was observed between time since treatment initiation and any of the assessed outcomes (Supplemental Information (SI−2)). No between-group difference or association analyses were performed for the 6-min walk test due to limited sample size. Mean standard scores, gross motor quotient, and bar, 95% CI, and percentage of children with impaired performance for each diagnostic group are shown in Table 3, and the distribution is illustrated in Fig. 3.

**Table 3:** Mean standard scores, gross motor quotient, bar, 95% CI, and percentage of children with impaired performance of the PDMS-2, the PEDI, and handgrip strength for each diagnostic group

	Children diagnosed with hematological cancers (PDMS-2 *n* = 32; PEDI *n* = 55; handgrip strength *n* = 15)		Children diagnosed with extracranial solid tumors (PDMS-2 *n* = 10; PEDI *n* = 15; handgrip strength *n* = 2)		Children diagnosed with central nervous system tumors (PDMS-2 *n* = 10; PEDI *n* = 13; handgrip strength *n* = 6)	
	Mean [95%CI]	Children scoring below average or worse (%)^#^	Mean [95%CI]	Children scoring below average or worse (%)^#^	Mean [95%CI]	Children scoring below average or worse (%)^#^
PDMS-2—Stationary (*n* = 44)	7.4 [6.3–8.5]	11/25 (44%)	6.9 [4.7–9.1]	5/10 (50%)	5.2 [3.2–7.2]	8/9 (89%)
PDMS-2—Locomotion (*n* = 42)	6.3 [5.2–7.5]	18/24 (75%)	5.4 [2.9–8.0]	7/9 (78%)	4.3 [2.6–6.1]	9/9 (100%)
PDMS-2—Object manipulation (*n* = 50)	8.2 [7.5–9.1]*	8/31 (26%)	7.3 [4.7–9.9]	4/10 (40%)	5.3 [3.0–7.7]*	7/9 (78%)
PDMS-2—Gross motor quotient (*n* = 37)	82.4 [75.9–88.8]	15/20 (75%)	79.6 [64.8–94.3]	6/9 (67%)	69.4 [56.9–81.8]	7/8 (88%)
PEDI – Mobility (functional) (*n* = 83) “below 10” = 10	22.7 [19.1–26.4]	38^▪^/55 (69%)	28.0 [17.6–38.3]	8^▪▪^/15 (53%)	19.4 [12.4–26.5]	11^▪▪▪^/13 (92%)
PEDI – Mobility (functional) (*n* = 83) “below 10” = 1	19.1 [14.5–23.8]		24.4 [11.9–36.8]		14.6 [4.9–24.3]	
PEDI – Mobility (caregivers’ ass.) (*n* = 83) “below 10” = 10	27.1 [23.8–30.4]	27^▪▪▪▪^/55 (49%)	30.9 [21.5–40.2]	6^▪▪▪▪▪^/15 (40%)	25.0 [16.0–33.9]	7^▪▪▪▪▪▪^/13 (54%)
PEDI – Mobility (caregivers’ ass.) (*n* = 83) “below 10” = 1	25.4 [21.4–29.4]		29.1 [18.3–39.9]		22.2 [11.3–33.1]	
Handgrip strength – right hand (*n* = 23)	0.19 [0.12–0.25]	11/15 (73%)	0.28 [−0.10–1.60]	2/2 (100%)	0.10 [0.02–0.19]	5/6 (83%)
Handgrip strength – left hand (*n* = 23)	0.17 [0.11–0.24]	12/15 (80%)	0.31 [−0.83–1.5]	2/2 (100%)	0.13 [0.04–0.23]	5/6 (83%)

^#^ For the PDMS-2, this equals a score ≤ the 16th percentile/−1 standard deviation score; for the PEDI, this equals a score ≤ −2 SDS; for handgrip strength, this equals a value ≤ −1 SDS

*Children diagnosed with central nervous system tumors perform significantly worse than children diagnosed with hematological cancers in the object manipulation domain (p = 0.02)

▪22 of the 38 children had a normative standard score “below 10”; ▪▪6 of the 8 children had a normative standard score “below 10”; ▪▪▪7 of the 11 children had a normative standard score “below 10”; ▪▪▪▪10 of the 27 children had a normative standard score “below 10”; ▪▪▪▪▪3 of the 6 children had a normative standard score “below 10”; ▪▪▪▪▪▪4 of the 7 children had a normative standard score “below 10”

## Discussion

This study shows that over two-thirds of preschool children (ages 1–5) newly diagnosed with cancer have below-average or worse gross motor function and functional level compared with age-expected norms. This aligns with a previous systematic review [10] that identified three studies – primarily including older children – reporting compromised gross motor function in children with newly diagnosed ALL [1, 19, 20]. Among these, Hartman et al. [19] is particularly relevant, as it assessed children aged 1–18 early in the disease trajectory, while the other studies included children aged ≥ 4 years [1, 20]. Using the Bayley Scales of Infant Development (BSID-II) and the Movement Assessment Battery for Children (MABC), Hartman et al. found significantly impaired gross motor function at diagnosis (mean −1.41 SDS) [19]. In our study, impairments were more pronounced (−2.11 SDS on the PDMS-2 gross motor quotient and −2.68 SDS on PEDI functional mobility). This may be explained by the developmental stage of the included children in the studies. Preschool years are critical for motor skill development, and disruption from illness, hospitalization, and treatment may have a stronger impact on younger than on older children, suggesting early disease affects motor development more profoundly in preschool-aged children. This is supported by a previous study on children with cancer, indicating that the youngest children have the most severe walking difficulties and loss of ability to support themselves on their legs during treatment [31]. Another explanation may be the inclusion of all cancer types in our study; children with extracranial solid tumors and central nervous system tumors demonstrated more impairment than children with hematological cancers. Notably, 9/13 children with central nervous system tumors had already had surgery for their tumor prior to the physical assessment, which may have influenced the result of the test. The timing of treatment initiation and the physical assessment may also influence our results. All but one child had initiated chemotherapy, with most of the children having initiated treatment with Vincristine and Dexamethasone at the time of the physical assessment. Vincristine is known to induce neuropathy, which can cause sensory impairments and reduced muscle function [4, 6], and treatment with Dexamethasone may lead to behavioral changes, including mood swings, aggression, and attention difficulties [32], which may limit the ability to complete or partake in the gross motor test. No difference was, however, found in the initiation of Vincristine or Dexamethasone between the completers and non-completers in our study.

Methodological differences may also contribute to the observed discrepancies between our results and the results of previous studies. The PDMS-2 may be considered more comprehensive (143 gross motor items) than the MABC (five gross motor tasks) and the BSID-II (72 gross motor items) [33, 34]. In group-based research, this can be a strength, as comprehensive tests often include more motor subdomains, thereby being more sensitive and detecting context-specific motor challenges. Conversely, a more comprehensive test increases the risk of failure due to fatigue, attention, or motivation, as reflected in the high non-completion rate in this study—a risk that can be further increased by Dexamethasone. Additionally, norm-referenced tests like the PDMS-2 are designed to interpret individual performance; using them for groups may limit interpretation at the group level unless distributional and contextual factors are also considered. In this study, we clarified the context for the comparison and included distributional factors, such as the full range of the sample and the percentage of children performing below average. High non-completion rates highlight testing challenges and introduce potential selection bias, possibly leading to over- or underestimation of results. Non-completion was mainly due to health issues, pain, or functional limitations, which may be linked to poorer motor function and thus is expected to underestimate impairment. Other reasons included logistics or low motivation, likely spanning the full range of motor ability.

Missing PDMS-2 data were anticipated during study planning, and PEDI was included to capture functional aspects of mobility. Results showed below-average functional mobility, indicating motor difficulties in over two-thirds of the study population. However, applying US-based PEDI norms to a Danish sample may be considered challenging due to cultural differences [25]. While functional mobility scores were similar, US children appeared more independent on the caregivers' assistance scale, likely reflecting contextual factors such as differences in home environments and changes in safety standards over time [25].

Although handgrip strength in our study could only be assessed in children aged 3 years and older and had a low completion rate, the results showed strength ≤ −1 SDS relative to the reference data for healthy children. Comparison with prior studies in children with cancer is limited, as they include only children aged 4 or above and use handheld dynamometers [1, 35–37]. Together with the very low completion rate of the 6-min walk test, in which only one child below 3 years could complete the test, this highlights the challenges of applying these outcomes to preschool children. A thorough discussion of the feasibility of the outcome measures has been published in another article [30], where results on the feasibility of the outcomes at 3 months and 6 months into treatment show that completion of the handgrip strength and 6-min walk test increases to 30% and 54%, respectively, at 6 months. Consequently, future studies should consider prioritizing outcome measures that are developmentally appropriate and feasible for preschool children during cancer treatment.

### Strengths and limitations

The key strength of this study is its exclusive focus on preschool-aged children, examining gross motor development, functional level, physical performance, and handgrip strength at cancer diagnosis. Including children with all cancer diagnoses extends the existing evidence base with a more heterogeneous and representative pediatric oncology sample; however, the small sample sizes within the handgrip strength subgroups and the limited number of participants completing the 6-min walk test limit the representativeness and generalizability of the findings. Additional strengths include the use of validated outcome measures, double data entry procedures, and the inclusion of relevant subgroup and sensitivity analysis. The single-site design may limit the generalizability of the findings, and finally, the interpretation of normative scores should also consider potential cross-cultural differences in motor development. Another limitation of this study is that the PDMS-2 has not been specifically examined in a Danish context, underscoring the need for caution when interpreting standardized motor test results applied to samples outside the original normative sample [38].

### Clinical implications

As medical treatment in most cases was initiated prior to the physical assessment, it is not possible to determine whether the motor deficits were pre-existing or attributable to the cancer or its treatment. However, regardless of their origin, the presence of motor deficits early in the treatment trajectory has important clinical implications. For preschoolers diagnosed with cancer, even for the children performing within or above the level expected for their age, offering and supporting opportunities for physical activity and active play during hospitalization is important to ensure continued support of gross motor development even during cancer treatment. Furthermore, ensuring early screening of all newly diagnosed preschool children as part of standard care could facilitate identification of children with reduced motor function and may facilitate timely referral to pediatric rehabilitation and support proactive interventions to preserve motor development and prevent further functional decline during treatment. While standardized assessments with acceptable psychometric properties are valuable in research, enabling objective measurement and comparison of motor outcomes across studies, the feasibility challenges observed in this study may limit their routine use in clinical practice for preschool children diagnosed with cancer. In the clinical setting, where the primary goal is to identify motor impairments and guide rehabilitation planning, functional assessments of a child's ability to perform age-appropriate everyday activities may yield more clinically meaningful information than standardized tools. In the present study, the domains in which the children experienced the greatest difficulties were the stationary domain (i.e., balancing) and the locomotion domain (i.e., moving from A to B in different ways), whereas object manipulation (i.e., throwing, catching, and kicking) was the domain with the fewest difficulties. In clinical practice, observations of activities such as walking along a corridor, balancing an obstacle course, or climbing stairs may offer a more feasible means of reflecting a child's functional abilities and rehabilitation needs in daily clinical practice.

Accordingly, identifying functional assessment tools appropriate for preschool children during cancer treatment, combined with systematic assessment of all children at treatment initiation, represents a promising approach for the early, clinically meaningful identification of rehabilitation needs.

## Conclusion

Preschool children newly diagnosed with cancer have below-average gross motor function, functional level, and muscle strength, showing clinically significant impairment. These findings highlight the need for systematic early-initiated targeted physical rehabilitation as an integrated part of cancer treatment for all preschool-aged children.

## Supplementary Information

Below is the link to the electronic supplementary material.

Supplementary file1 (DOCX 107 KB)

Supplementary file2 (DOCX 106 KB)

## Data Availability

The datasets generated and/or analyzed during the current study are not publicly available due to Danish and EU personal data legislation but are available from the corresponding author upon reasonable request.

## Abbreviations

PDMS-2: Peabody Developmental Motor Scales–Second Edition
PEDI: Pediatric Evaluation of Disability Inventory
SDS: Standard Deviation Score
BSID-II: Bayley Scales of Infant Development
MABC: Movement Assessment Battery for Children
