# Supplementary material for: Gross motor function, functional level, physical performance, and muscle strength in preschool children newly diagnosed with cancer: Data from the RePlay trial
Source: Eur J Pediatr. 2026 Aug 18;185(9):674. doi: 10.1007/s00431-026-07333-3 (PMC13481491; doi:10.1007/s00431-026-07333-3)
Supplement: Supplementary file 1 [file 431_2026_7333_MOESM1_ESM.docx]

|  | **Hematological Cancer vs Extracranial Solid tumor** | **p-value** | **Hematological Cancer vs Central Nervous System Tumor** | **p-value** | **Central Nervous system Tumor vs Extracranial Solid Tumor** | **p-value** |
| --- | --- | --- | --- | --- | --- | --- |
| PDMS-2 Stationary | -0.83(-2.95 to 1.28) | 0.43 | -1.86 (-4.04 to 0.33) | 0.09 | 1.02 (-1.73 to 3.78) | 0.46 |
| PDMS-2 Locomotion | -1.06 (-3.44 to 1.31) | 0.37 | -1.95 (-4.20 to 0.30) | 0.09 | 0.89 (-1.99 to 3.77) | 0.54 |
| PDMS-2 Object manipulation | -1.54 (-3.56 to 0.49) | 0.13 | **-2.56 (-4.61 to -0.51)** | **0.02** | 1.02 (-1.64 to 3.69) | 0.44 |
| PDMS-2 Gross Motor Quotient | -5.13 (-18.37 to 8.11) | 0.43 | -11.28 (-24.71 to 2.15) | 0.10 | 6.15 ( -10.71 to 23.01) | 0.46 |
| PEDI Mobility (functional)  ”Below 10” = 10 | 5.97 (-2.36 to 14.29) | 0.16 | -4.44 (-13.35 to 4.46) | 0.32 | 10.41 (-0.69 to 21.52) | 0.06 |
| PEDI Mobility (functional)  ”Below 10” = 1 | 6.11 (-4.43 to 16.66) | 0.25 | -5.91 (-17.18 to 5.36) | 0.30 | 12.03 (-2.04 to 26.08) | 0.09 |
| PEDI Mobility (caregivers’ ass.)  ”Below 10” = 10 | 4.68 (-3.17 to 12.53) | 0.24 | -3.41 (-11.80 to 4.98) | 0.42 | 8.09 (-2.38 to 18.55) | 0.13 |
| PEDI Mobility (caregivers’ ass.) ”Below 10” = 1 | 4.71 (-4.69 to 14.10) | 0.32 | -4.84 (-14.89 to 5.20) | 0.34 | 9.54 (-2.98 to 22.06) | 0.13 |

**Supplemental Information (SI-1)**
Between group comparison for the PDMS-2 and the PEDI scores.
