# Supplementary material for: Gross motor function, functional level, physical performance, and muscle strength in preschool children newly diagnosed with cancer: Data from the RePlay trial
Source: Eur J Pediatr. 2026 Aug 18;185(9):674. doi: 10.1007/s00431-026-07333-3 (PMC13481491; doi:10.1007/s00431-026-07333-3)
Supplement: Supplementary file 2 [file 431_2026_7333_MOESM2_ESM.docx]

| **Outcome** | **β (95% CI)** | **p-value** |
| --- | --- | --- |
| PDMS-2 Stationary | 0.11(-0.03 to 0.25) | 0.13 |
| PDMS-2 Locomotion | 0.03 (-0.12 to 0.18) | 0.66 |
| PDMS-2 Object manipulation | 0.14 (-0.004 to 0.28) | 0.06 |
| PDMS-2 Gross Motor Quotient | 0.63(-0.24 to 1.51) | 0.15 |
| PEDI Mobility (functional) | -0.22 (-0.71 to 0.28) | 0.38 |
| PEDI Mobility (caregivers’ ass.) | -0.29 (-0.76 to 0.17) | 0.21 |
| Handgrip strength – right hand | -0.004 (-0.02 to 0.01) | 0.47 |
| Handgrip strength – left hand | 0.00 (-0.01 to 0.01) | 0.90 |

**Supplemental Information (SI-2)**
Association with time since treatment initiation for the PDMS-2, the PEDI, and the handgrip strength scores
